# Growth patterns of survivors of retinoblastoma treated with ophthalmic artery chemosurgery

**DOI:** 10.1371/journal.pone.0197052

**Published:** 2018-05-07

**Authors:** Sruti S. Akella, Jasmine H. Francis, Andrea Knezevic, Irina Ostrovnaya, Y. Pierre Gobin, Danielle Friedman, Edith Guarini, Lindsey Eibeler, Federica Catalanotti, David H. Abramson

**Affiliations:** 1 Transitional Year Intern, Department of Medicine, Memorial Sloan Kettering Cancer Center, New York, New York, United States of America; 2 Department of Surgery, Ophthalmic Oncology Service Memorial Sloan Kettering Cancer Center, New York, New York, United States of America; 3 Department of Ophthalmology, Weill-Cornell School of Medicine, New York, New York, United States of America; 4 Department of Epidemiology and Biostatistics, Memorial Sloan Kettering Cancer Center, New York, New York, United States of America; 5 Department of Radiology and Neurosurgery, New York Presbyterian Hospital, New York, New York, United States of America; 6 Department of Pediatrics, Memorial Sloan Kettering Cancer Center, New York, New York, United States of America; Massachusetts Eye & Ear Infirmary, Harvard Medical School, UNITED STATES

## Abstract

Although studies from pediatric cancers (largely acute lymphoblastic leukemia) have shown that patients undergoing systemic chemotherapy may experience decreased growth velocity during the treatment phase, no such data exist for retinoblastoma patients treated with systemic chemotherapy or ophthalmic artery chemosurgery (OAC). The purpose of this study is to report growth patterns of our retinoblastoma (Rb) population who were treated with OAC in a retrospective, single center (Memorial Sloan Kettering Cancer Center) review of 341 patients treated between 2006 and 2016. Children who only received OAC were classified as naive; those who were treated initially with systemic chemotherapy and subsequently presented to our center for OAC were termed secondary; and a small group of patients who received single-agent systemic chemotherapy prior to OAC were labeled bridge. For all patients, height and weight were recorded at monthly intervals during OAC (short-term) and then annually during a follow-up period (long-term) up to 3 years after treatment. Excluded from this study were children who received external radiation therapy and those with genetic syndromes, which are independently associated with growth derangements. During OAC, there was no significant difference in growth velocity between the naïve and secondary groups. In either group, number of treatments also did not affect growth rate. Three years after the end of OAC, naïve patients were in the 68^th^ percentile by height (95% CI 61.30, 74.63) compared to secondary patients in the 61^st^ percentile (95% CI 51.1, 71.47). Both groups were in the same weight percentiles during the first two years of follow-up but at the three-year follow-up period, naïve patients were in the 63^rd^ percentile (95% CI 57.4, 69.4) and secondary patients were in the 60^th^ percentile (95% CI 50.4, 69.7). OAC for retinoblastoma does not appear to impact short-term growth velocity, weight gain during the treatment period or after three years.

## Introduction

Retinoblastoma is the most common primary intraocular malignancy in children, and recent therapeutic advances have made it one of the most curable forms of childhood cancer, with survival rates exceeding 95% in some countries [1]. The increasing number of survivors of childhood retinoblastoma has led to a growing interest in the long-term adverse effects of treatment.

The incidence of childhood cancers, including retinoblastoma, coincides with periods of rapid skeletal growth [2]. There are no data, however, to our knowledge, regarding the potential incidence of growth impairment in survivors of retinoblastoma treated with systemic chemotherapy or OAC, which may or may not impact height. Several studies [3,4,5] in the literature have reported long-term sequela of survivors of retinoblastoma but none have commented on growth patterns.

During the duration of systemic chemotherapy for other pediatric cancers, early growth deceleration with bone age retardation and focal radiological findings have previously been documented [6]. In patients with acute lymphoblastic leukemia receiving systemic chemotherapy, decreased growth velocity has been reported during all phases of treatment [7–9]. However, nearly 70% of these patients will show a variable degree of catch-up growth depending on the type and dosage of the chemotherapy regimen administered [10]. Until the introduction of ophthalmic artery chemosurgery (OAC), systemic chemotherapy (usually with carboplatin, vincristine, and etoposide) was used for intraocular retinoblastoma [11,12] since the abandonment of external beam radiation. OAC uses approximately one-twentieth of the typical total systemic dose [12] and as a result has far fewer and less intense systemic side effects compared to intravenous administration [13,14]. The purpose of this study is to report growth patterns in a large cohort of retinoblastoma survivors who were treated using OAC.

## Materials and methods

A retrospective survey of all patients treated with OAC between May 1, 2006, and October 1, 2016 at Memorial Sloan Kettering Cancer Center was performed. Excluded from the study were children with known genetic syndromes, including Down syndrome and 13q deletion and children who had received external beam radiation therapy, which are both independently associated with short stature. Patients were categorized in one of three groups: those who received OAC as the primary treatment (naive); those who received OAC after systemic therapy (secondary); and patients who received a round of single-agent systemic chemotherapy (bridge) prior to treatment with OAC.

For every child in each group, height and weight were obtained retrospectively at first office visit, at every subsequent OAC treatment (short-term), and annually at 1, 2 and 3 years post-chemotherapy (long-term), where data was available. Baseline was defined as measurements taken at first OAC treatment. Height was measured using a length board for infants and a scale-mounted stadiometer for older children. Additional details were recorded about tumor laterality and total cumulative doses of carboplatin, topotecan, and melphalan. A total of 341 patients with 372 OAC sessions were included. This study was approved by the institutional review board of Memorial Sloan Kettering Cancer Center. All data were de-identified and analyzed anonymously.

Monthly height and weight measurements from the OAC treatment period were used to calculate growth velocities for each patient, which were compared between chemotherapy groups using the univariate ANOVA test. For long term growth analysis, height and weight percentiles were compared between groups at years 1, 2 and 3 after the start of treatment. Multivariate ANOVA models were adjusted for age, baseline measurement, and number of treatments as covariates in addition to chemotherapy group.

Height and weight percentiles were calculated for each child based on sex and age using World Health Organization (WHO) growth curves for ages up to 24 months and Centers for Disease Control and Protection (CDC) growth curves for ages greater than 24 months.

## Results

Demographics of our population are shown in Table 1. Naïve patients received treatment an average of two months younger than secondary patients (16 versus 18 months: p = 0.006), and at first treatment were shorter (p<0.001) and weighed less (p = 0.001). Bridge patients began treatment at age 5 months, and were predictably smaller than patients in other groups. Patients in all groups received a median number of three OAC treatments (p = 0.23).

**Table 1:** Demographic and clinical characteristics, overall and by chemotherapy group.

	Overall (n = 341)	Bridge (n = 27)	Secondary (n = 153)	Naïve (n = 161)	P
Female sex n(%)	182 (53.4)	17 (63.0)	85 (55.6)	79 (49)	0.29
Age at first treatment (months)–median (range)	16 (2, 122)	5 (2, 52)	18 (4, 123)	16 (3, 117)	0.006
Less than 2 years of age	245 (71.9)	23 (85.2)	110 (71.9)	112 (69.6)	0.25
Height at first treatment* (cm)–median (range)	79 (58, 138)	65 (59, 97)	82 (60, 138)	79 (58, 134)	<0.001
Weight at first treatment† (kg)–median (range)	10.5 (5.6, 39.5)	7.5 (5.7, 16.1)	11.0 (6.0, 35.4)	10.5 (5.6, 39.5)	0.001
Bilateral retinoblastoma	150 (44)	15 (55.6)	81 (52.9)	54 (33.5)	0.001
Number of treatments‡ –median (range)	3 (1, 11)	3 (1, 7)	3 (1, 11)	3 (1, 9)	0.23
Chemotherapy drugs‡					
Melphalan	335 (98.2)	26 (96.3)	151 (98.7)	158 (98.1)	0.52
Topotecan	283 (83.0)	19 (70.4)	131 (85.6)	133 (82.6)	0.15
Carboplatin	263 (77.1)	16 (59.3)	127 (83.0)	120 (74.5)	0.01
Methotrexate	2 (0.6)	1 (3.7)	1 (0.7)	0	0.08

Values report as frequent (percent), unless otherwise noted.

Group comparison performed with Chi-square test for categorical values and ANOVA for continuous variables. Fisher’s exact test used for Melphalan and Methotrexate due to small expected cell count. Total of 341 patients (372 treatment periods); 27 patients received more than none treatment.

*Available for 292 patients

†Available for 335 patients

‡ All chemotherapy data reported on last chemotherapy treatment period for each patient.

### Short-term growth analysis

**Fig 1:**
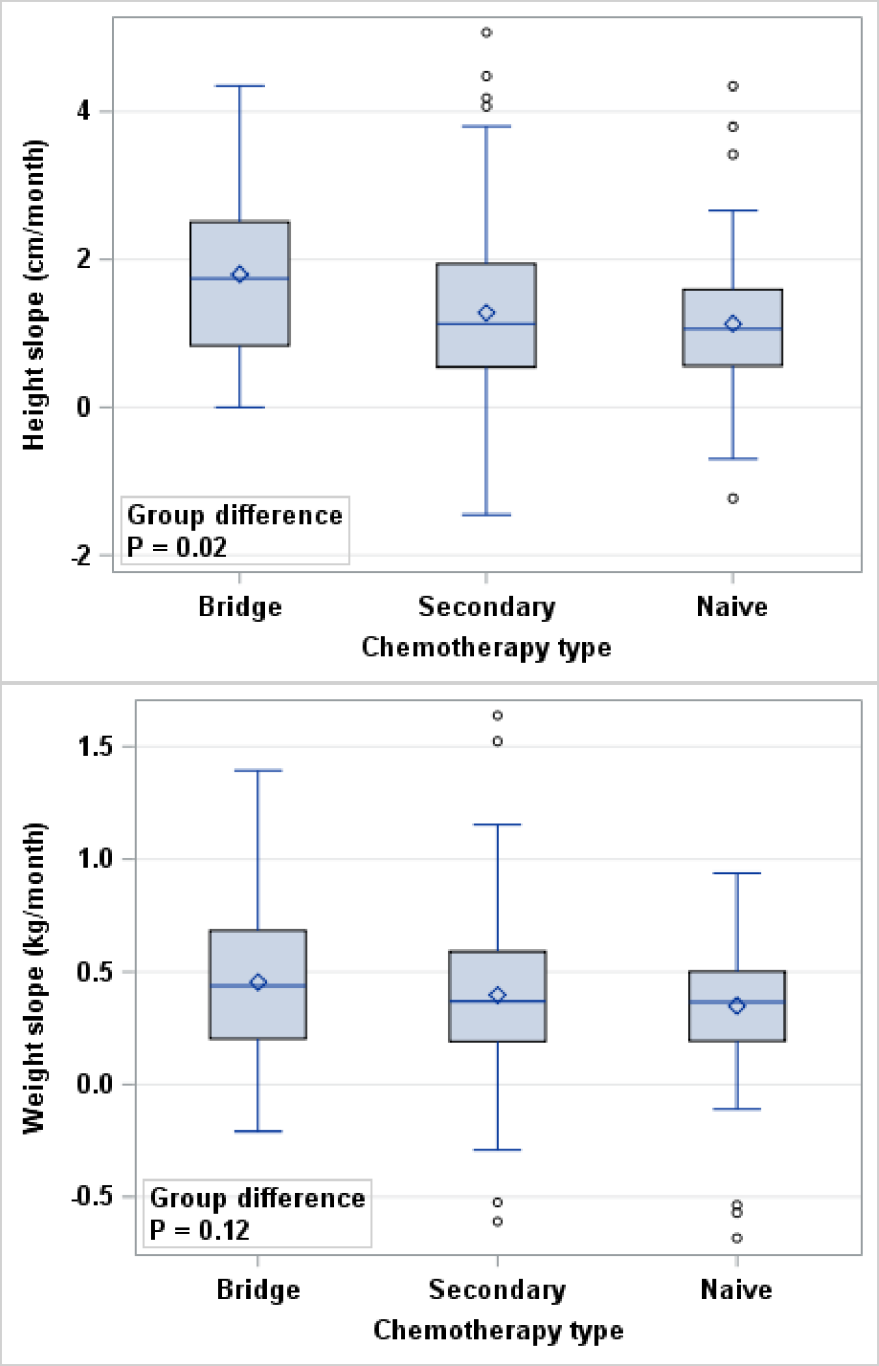
Growth velocity by height and weight during OAC. Growth velocity for height (n = 255) and weight (n = 324) during treatment by chemotherapy group. Growth slope is calculated starting at first treatment and using height and weight measurements up to time of treatment number 4 (or last available measurement prior to treatment 4); slope is calculated for patients who have a measurement available at first treatment and at least one follow-up. Mean height growth is significantly different between groups (ANOVA p = 0.02) with the mean for the bridge group significantly higher than the mean for the naïve group (mean difference: 0.66 cm/month; 95% CI: 0.10, 1.21; p = 0.01). Mean weight growth is not significantly different by group (ANOVA p = 0.12).

During OAC, bridge patients grew fastest by height (1.80 centimeters per month; 95% CI 1.38, 2.22), followed by secondary patients (1.29 cm/month; 95% CI 1.11, 1.47) and finally naïve patients (Fig 1, Table 2). These group differences in linear growth velocity are significantly different in univariate analysis (ANOVA p = 0.02). However, in pairwise comparisons only the mean difference in linear growth velocity between bridge and naïve patients was significant (Table 2). Mean change in weight was not significantly different by group in univariate analysis (ANOVA p = 0.12) (Fig 1).

**Table 2:** Height and weight growth by group and group difference.

**Univariate models**	**Height**	**Weight**
	*Growth slope (cm/month)*	*Growth slope (kg/month)*
*Mean (95% CI)*, *N*		
Bridge	1.80 (1.38, 2.22), 24	0.45 (0.35, 0.55), 39
Naïve	1.14 (0.95, 1.33), 116	0.35 (0.30, 0.40), 157
Secondary	1.29 (1.11, 1.47), 126	0.40 (0.35, 0.45), 147
*Mean difference (95% CI)*, *P*		
Bridge–Naïve	**0.66 (0.10, 1.21), 0.02**	0.11 (-0.03, 0.24), 0.14
Bridge–Secondary	0.51 (-0.04, 1.06), 0.08	0.06 (-0.08, 0.19), 0.57
Secondary–Naïve	0.15 (-0.17, 0.47), 0.51	0.05 (-0.04, 0.13), 0.37
**Multivariate models**	**Height**	**Weight**
	*P-value of model predictors*	*P-value of model predictors*
Type of chemotherapy	0.40	0.27
Age at baseline	<0.001	0.93
Height/weight at baseline	<0.001	0.57
Number of treatments	0.31	0.18

When assessing independent predictors of growth outcomes in multivariate models, we found that type of chemotherapy is not an important predictor for growth velocity in height (p = 0.40) nor weight (p = 0.27) during the OAC treatment period (Table 2). Age at baseline is an important predictor of growth velocity in height (p<0.001) but not weight (p = 0.93). The total number of treatments received during OAC does not affect growth velocity in height (p = 0.31) nor weight (p = 0.18).

### Three-year growth analysis

**Fig 2:**
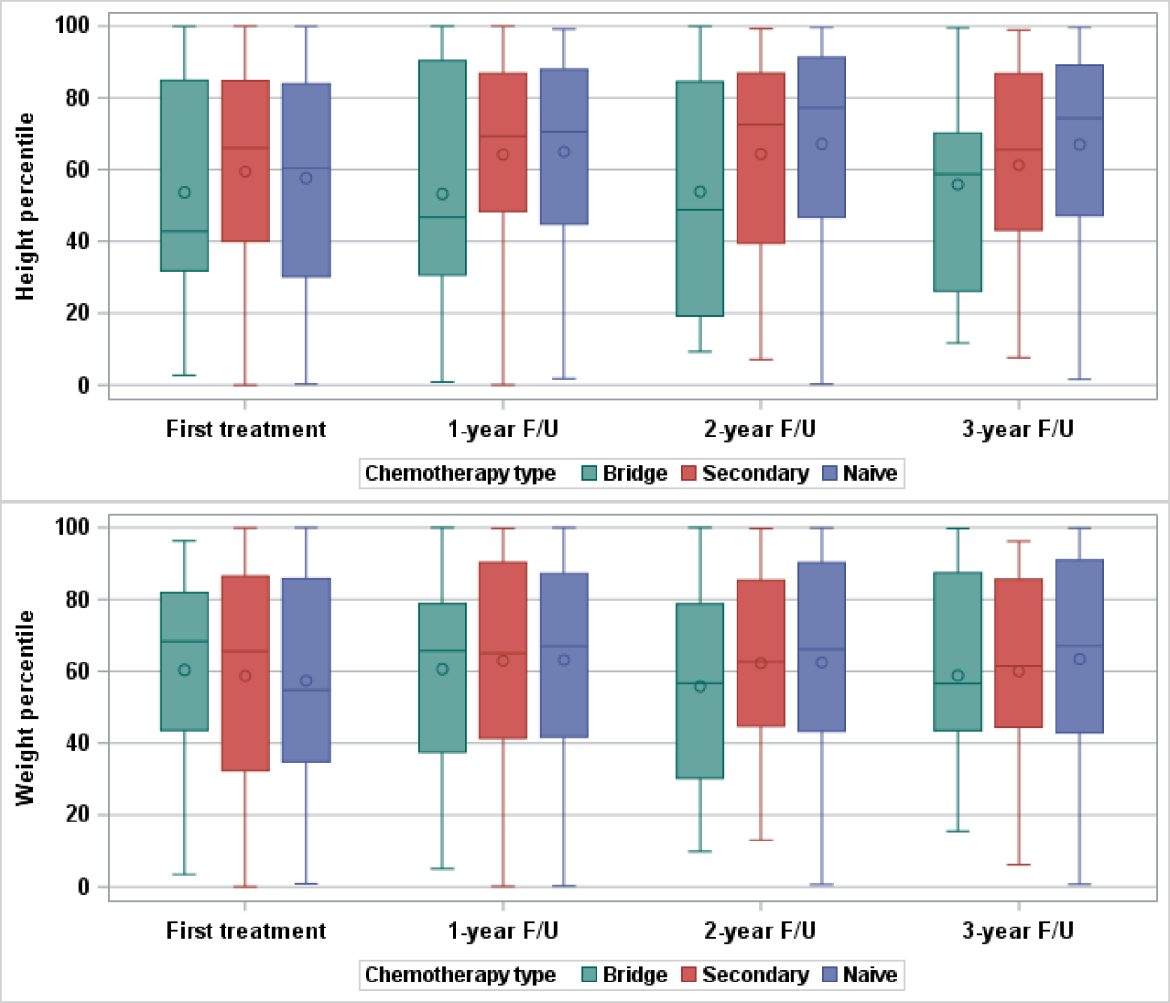
Height and weight percentiles at first treatment, 1, 2 and 3 year follow-up by chemotherapy group. Mean height percentiles are not significantly different by group at first treatment (ANOVA p = 0.72, n = 291), 1 year (p = 0.14, n = 276), 2 year (p = 0.06, n = 203) and 3 year (p = 0.49, n = 134) follow-up. Mean weight percentiles are also not significantly different by group at first treatment (p = 0.86, n = 334), 1 year (p = 0.93, n = 290), 2 year (p = 0.65, n = 209) and 3 year (p = 0.73, n = 137) follow-up.

Annually in the three-year follow-up period, naïve patients were consistently in a higher height percentile than secondary patients, although no group differences were significantly different (Fig 2, Table 3). At two years of follow-up, naïve patients were in the 68^th^ percentile on average, compared to the 63^rd^ percentile for secondary patients (mean difference: 4.6 percentile points, 95% CI: -5.7, 14.9). No clear trend was seen when comparing weight percentiles. The proportion of patients who dropped more than 10 percentile points in height at 1, 2 and 3 years compared to baseline is higher in the secondary group compared to the naïve group (Table 4). At two years of follow-up, 13.0% of naïve patients had dropped more than 10 percentile points in height, compared to 21.7% in the secondary group. No clear trend was seen when comparing drop in weight percentile.

**Table 3:** Height and weight percentile by group and group difference.

	Baseline	1 year	2 years	3 years
*Height percentile mean (95% CI)*, *N*				
**Bridge**	54 (39, 68), 17	54 (43, 65), 25	53 (41, 65), 23	56 (43, 69), 18
**Naïve**	58 (53, 63), 138	66 (61, 70), 135	68 (63, 73), 113	65 (59, 71), 83
**Secondary**	60 (54, 65), 136	65 (60, 70), 116	63 (57, 70), 67	62 (52, 71), 33
*Height mean difference (95% CI)*				
**Bridge–Naïve**	-4.0 (-22.5, 14.4)	-12.0 (-26.3, 2.3)	-15.0 (-30.3, 0.3)	-8.5 (-25.7, 8.7)
**Bridge–Secondary**	-5.9 (-24.3, 12.6)	-11.1 (-25.6, 3.4)	-10.4 (-26.6, 5.8)	-5.6 (-25.0, 13.8)
**Naïve–Secondary**	-1.8 (-10.5, 6.8)	0.9 (-7.4, 9.2)	4.6 (-5.7, 14.9)	2.9 (-10.7, 16.6)
*Weight percentile mean (95% CI)*, *N*				
**Bridge**	60 (49, 71), 27	61 (50, 72), 26	56 (44, 67), 24	59 (46, 72), 19
**Naïve**	57 (53, 62), 158	63 (58, 68), 145	62 (56, 67), 116	62 (56, 68), 85
**Secondary**	59 (54, 63), 149	63 (58, 68), 119	61 (55, 68), 69	58 (49, 68), 33
*Weight mean difference (95% CI)*				
**Bridge–Naïve**	2.9 (-11.4, 17.3)	-2.3 (-16.6, 12.0)	-5.9 (-21.1, 9.3)	-3.6 (-20.6, 13.4)
**Bridge–Secondary**	1.6 (-12.8, 16.1)	-2.1 (-16.7, 12.4)	-5.6 (-21.6, 10.5)	0.6 (-18.7, 19.8)
**Naïve–Secondary**	-1.3 (-9.2, 6.6)	0.1 (-8.2, 8.4)	0.4 (-9.9, 10.7)	4.2 (-9.6, 17.9)

**Table 4:** Proportion of height and weight percentile drop >10% from baseline by group.

	1 year	2 years	3 years
*Height percentile drop >10% No*. *(%)*			
**Bridge**	5 (31.3%)	5 (31.3%)	2 (18.2%)
**Naïve**	18 (15.4%)	12 (13.0%)	11 (17.2%)
**Secondary**	25 (22.9%)	13 (21.7%)	8 (29.6%)
*Weight percentile drop >10% No*. *(%)*			
**Bridge**	8 (30.8%)	12 (50.0%)	7 (36.8%)
**Naïve**	28 (19.7%)	23 (20.2%)	18 (21.7%)
**Secondary**	21 (18.0%)	15 (22.1%)	9 (28.1%)

## Discussion

As the survival rates of retinoblastoma begin to exceed 95%, the late-effects of cancer treatment have obtained increased attention, and the long-term monitoring of survivors has become an important part of their overall health care. In addition, the understanding of long term side effects drives management. External beam irradiation was the only way advanced eyes were salvaged for most of the 20^th^ century but, because of its impact on the development of secondary cancers, years later the technique has been abandoned worldwide [1].

Several studies have reported on psychological [15,16], behavioral [17,18], and functional outcomes [19,20] in adult survivors of retinoblastoma. While many papers report on second cancer incidence, patterns and outcomes for retinoblastoma patients, only a few [4,5,21] have reported long-term treatment-related medical conditions of retinoblastoma survivors. Suzuki et al. [4] retrospectively evaluated the long-term prognosis of intra-arterial therapy (in 408 eyes of 343 retinoblastoma patients) over a 20-year time period, but they did not report on growth outcomes. Friedman et al [5] modeled the Childhood Cancer Survivor Study and reported medical outcomes and general health of 470 adult survivors of retinoblastoma with a median follow-up of 42 years. They found that survivors of retinoblastoma were 1.4 times more likely to report any chronic condition, and 7.6 times more likely to have a severe or life-threatening chronic condition when compared with a similarly aged cohort of individuals without a history of retinoblastoma. They do not report on growth parameters during the chemotherapy administration period or the subsequent follow up period.

Acute lymphoblastic leukemia (ALL) survivors currently form the largest group of long-term survivors from childhood cancer, and treatment of ALL with chemotherapy alone during childhood has been associated with final height deficits [22,23]. The mechanism by which chemotherapy exerts its influence on growth is unclear. Several authors [23,24] have hypothesized that treatment induces hypothalamic-pituitary abnormalities ultimately leading to growth hormone (GH) deficiency.

In our study, data were collected retrospectively and we did not investigate GH secretion or pubertal status in our population. In addition, the sample size of bridge patients was too small to draw any significant conclusions and we did not pursue analysis. Their rapid growth during OAC is likely attributed to their younger age, consistent with published growth curves showing that younger infants have higher rates of linear growth [25].

Our secondary group appeared to grow more rapidly than our naïve group during treatment with OAC, but this difference was not found to be statistically significant. Both groups are above the 50^th^ percentile for linear growth. Prior studies on systemic chemotherapy [26] have documented a “catch-up growth” phenomenon in which the cessation of intravenous chemotherapy coincides with resumption of growth and an increase in growth markers above pre-chemotherapy levels. We may hypothesize that additional treatment with OAC does not suppress this period of accelerated growth, in contrast to a previous study [27] in which a third phase of systemic chemotherapy inhibited catch-up growth. Our lack of statistical power may be related to a small sample size.

We also found that in the three-year follow-up period, both naïve and secondary patients were consistently taller than same-age children in the general population, suggesting that OAC does not affect growth whether given as primary treatment or after systemic chemotherapy, in contrast to a recent study [28] in which 26% of pediatric cancer patients (30% with retinoblastoma) treated with only systemic chemotherapy had a height less than the third percentile for that age. However, we did not have the opportunity to serially measure growth during systemic chemotherapy because we abandoned systemic chemotherapy for intraocular retinoblastoma more than 11 years ago (with the exception of bridge patients) [29].

Although a previous study [30] has reported on the prevalence of childhood obesity in pediatric cancer survivors treated with systemic chemotherapy, no such effect was seen in our naïve group at the three-year follow-up period.

The introduction of OAC has resulted in a dramatic increase in ocular retention rate without compromising patient retinoblastoma survival [31], but as with any novel therapy, long-term effects must be carefully monitored. OAC does not appear to affect linear growth during treatment, and furthermore does not appear to inhibit catch-up growth after systemic chemotherapy. OAC is not associated with short stature long-term. Further longitudinal studies are needed to assess final stature in adulthood.
